# *Ulva prolifera* Stress in the Yellow Sea of China: Suppressed Antioxidant Capacity and Induced Inflammatory Response of the Japanese Flounder (*Paralichthys olivaceus*)

**DOI:** 10.3390/ani13243768

**Published:** 2023-12-06

**Authors:** Dan Xu, Yongzheng Tang, Wenlong Li, Yingming Yang

**Affiliations:** 1 School of Ocean, Yantai University, Yantai 264005, China; xudan@ytu.edu.cn (D.X.); 13906380063@163.com (Y.T.); 2 Key Laboratory for Sustainable Development of Marine Fisheries, Ministry of Agriculture and Rural Affairs, Yellow Sea Fisheries Research Institute, Chinese Academy of Fishery Sciences, 106 Nanjing Road, Qingdao 266071, China; liwl@ysfri.ac.cn; 3 Laboratory for Marine Fisheries Science and Food Production Processes, Qingdao Laoshan Laboratory for Marine Science and TechnFology, Qingdao 266235, China

**Keywords:** *Ulva prolifera*, growth performance, antioxidant capacity, inflammatory response, *Paralichthys olivaceus*

## Abstract

**Simple Summary:**

With the aggravation of water eutrophication and global climate change, macroalgal blooms along coastal areas worldwide have become increasingly common in recent years. As the largest green macroalgal bloom, in the Yellow Sea of China we can observe that environmental stress caused by the overgrowth and degradation of *Ulva prolifera* has a harmful effect on marine organisms and the aquaculture industry. Therefore, exploring the harmful effect of *Ulva prolifera* stress on fish could promote the development of aquaculture in the Yellow Sea. This study revealed that *Ulva prolifera* stress suppressed antioxidant capacity in the liver and induced an inflammatory response accompanied by the activation of endoplasmic reticulum stress in the head kidney of the Japanese flounder, which could lead to huge damage to fish health.

**Abstract:**

As the largest green macroalgal bloom in the Yellow Sea of China, the overgrowth and degradation of *Ulva prolifera* (*U*. *prolifera*) have a harmful effect on marine organisms and the aquaculture industry. However, the regulation mechanism of *U. prolifera* stress on the antioxidant capacity and inflammatory response of marine fish is still not completely understood. A 15-day exposure experiment was conducted to evaluate the effects of *U. prolifera* stress on the antioxidant capacity and inflammatory response of the Japanese flounder (*Paralichthys olivaceus*) (283.11 ± 6.45 g). The results showed that *U*. *prolifera* stress significantly decreased their survival rate. Serum total antioxidant capacity (T-AOC) and non-specific immune-related enzyme activities were significantly impacted under *U*. *prolifera* conditions. Moreover, *U*. *prolifera* stress significantly decreased T-AOC, superoxide dismutase (SOD) activity, and catalase (CAT) activities in the liver, while malondialdehyde (MDA) contents were significantly increased. Similarly, antioxidant-related gene (*cat*, *nrf2,* and *keap1*) expressions were synchronously downregulated in the liver under *U*. *prolifera* stress. Furthermore, *U*. *prolifera* stress significantly upregulated pro-inflammatory gene (*tnf-α*, *il-1β*, *ifn-γ*, and *p65*) expressions and the phosphorylation levels of the p38 and JNK MAPK pathways in the head kidney. In addition, endoplasmic reticulum (ER) stress-related gene and protein expressions were also upregulated in the head kidney. Overall, these results revealed that *U. prolifera* stress suppressed the antioxidant capacity and induced an inflammatory response in the Japanese flounder. This study could advance the understanding of the adverse effects of *U*. *prolifera* stress on marine benthic fish and promote the sustainable development of aquaculture.

## 1. Introduction

In recent years, due to the escalation in water eutrophication and the impact of global climate change, there has been a substantial rise in the occurrence of macroalgal blooms along coastal regions worldwide [1,2,3]. The largest green macroalgal bloom in the Yellow Sea off the Shandong Province coast of China, which is mainly caused by *Ulva prolifera* (*U. Prolifera*), has existed since 2007, exceeding 1 million tons of production every year [4,5]. *U. prolifera*, although not toxic, can cause severe harm to the environment and marine organisms when it accumulates in large quantities due to the possible decomposition of organic materials following the algal bloom phase [6,7]. Among them, the death of fish caused by *U. prolifera* outbreaks always leads to an enormous economic loss to the aquaculture industry of the Yellow Sea [8,9]. Therefore, exploring the harmful effects of *U. prolifera* stress and degradation on fish could promote the development of aquaculture in the Yellow Sea.

Previous studies have proven that massive blooms of macroalgae in a vibrant shade of green can disrupt the delicate equilibrium of coastal ecosystems, influencing the intricate exchanges between air and sea and causing deterioration in the quality of the seawater [10,11]. The death of fish caused by green macroalgal bloom stress is mainly caused by hypoxia, acidification, and the presence of toxic substances [6]. It is known that those environmental stresses can result in the accumulation of detrimental ROS compounds, triggering signaling cascades and potentially harming proteins, lipids, and DNA if the organism is unable to mitigate the accrual of ROS, leading to cellular oxidation [12]. The antioxidant and immune defense mechanisms play an important role in resisting various environmental stressors. The fish antioxidant system balances oxidation and anti-oxidation processes through several antioxidant enzymes, such as superoxide dismutase (SOD), catalase (CAT), and other small molecular substances [13,14]. Oxidative stress is caused by an imbalance in the production and detoxification of reactive oxygen species within cells. Previous studies in fish have shown that environmental pollution can induce oxidative stress [15,16]. Furthermore, apart from the antioxidant system, the immune system is also critical for fish to resist external stimulation in the aquatic environment [17,18]. Fish, due to their less-evolved adaptive immune function, are more reliant on their innate immunity [19]. As a unique organ for teleost fish, the head kidney has an important immune function [20]. Environmental stimulation can activate pathogen recognition receptors and then regulate inflammatory signaling cascades, such as mitogen-activated protein kinase (MAPK) and nuclear transcription factor kappa-B (NF-κB) pathways [21]. However, the regulation mechanism of *U. prolifera* degradation on antioxidant capacity and inflammatory response in marine fish is still not completely understood and needs further investigation.

The Japanese flounder (*Paralichthys olivaceus*) is an economically important carnivorous marine flatfish species distributed in the Yellow Sea of China. As a marine carnivorous flatfish, the Japanese flounder is sensitive to various forms of environmental marine pollution, such as green macroalgal blooms, persistent organic pollutants, and heavy metal contamination [22]. However, there is a lack of systematic studies about the effects of *U. prolifera* degradation on growth performance and anti-stress capabilities in flatfish. Therefore, this study aimed to investigate the effects of *U*. *prolifera* stress on the antioxidant capacity and inflammatory response of the Japanese flounder. This study could advance the understanding of the adverse effects of *U*. *prolifera* stress on marine benthic fish and promote the sustainable development of aquaculture.

## 2. Material and Methods

In the present study, all experiments conducted on the fish were strictly adhered to the Laboratory Animals Management Rule (Chinese Order No. 676 of the State Council, revised 1 March 2017).

### 2.1. Fish Acclimation and Exposure Experiment

Healthy Japanese flounder (initial average weight of 283.11 ± 6.45 g) were obtained from Yellow Sea Aquatic Product Co., Ltd. (Yantai, China) and the subsequent experiment was carried out only on fish obtained from the company. Prior to beginning the experiment, the Japanese flounder were cultured in the conventional seawater (salinity: 28–33 ‰; dissolved oxygen: 5.5–7.0 mg/L; water temperature: 22–24 °C; pH: 7.30–7.65) for 2 weeks to adapt to the aquaculture environment fed with suited commercial feed. After 2 weeks of acclimation, the Japanese flounder were randomly distributed to three replicates per treatment in the fiberglass tanks (volume = 600 L) with each tank housing 30 fish.

The seawater contained macroalgae *U. prolifera* was collected from Haiyang Beach (Yellow Sea, Yantai, China) during the bloomed period (the end of June and August). Then, the collected seawater was only filtered through a 100 μm mesh to remove the remaining *U. prolifera* particulates and stored in the prepared tank (d = 250 cm; h = 120 cm; volume = 6000 L) for further exposure experiments. The Japanese flounder were then exposed and cultured in the prepared water environment of *U*. *prolifera* (the UP group) for 15 days with half of the water being replaced daily. The flounder cultured in normal seawater without *U*. *prolifera* pollution maintained through the recirculating aquaculture system (filtration, disinfection, and chemical processing) was considered as the control group (CON) (Figure S1). During the experiment, the control and UP group were subjected to the same water temperature and dissolved oxygen levels. No food was provided during the experiment. Additionally, the physiological condition of the fish was examined daily, and any instances of mortality were duly recorded.

### 2.2. Sample Collection

After concluding the experiment, the fish were anesthetized using MS-222 (1:10,000; Sigma, St. Louis, MO, USA) before sample collection. Blood samples were extracted from the caudal vein of six fish per tank and kept at 4 °C for 12 h. To analyze the enzyme activity, the serum was separated from the residual blood cells through centrifugation (1500× *g*/10 min) and stored at −80 °C. The liver and head kidney tissues were randomly selected from four fish per tank, thoroughly washed with phosphate buffer, and then stored into the 1.5 mL Eppendorf tubes (RNase-Free, Axygen, San Francisco, CA, USA) in liquid nitrogen. All specimens were maintained at −80 °C for subsequent evaluation of enzyme activity assessments and analysis of gene and protein expressions.

### 2.3. Enzyme Activity Assays in Serum and Tissue

For the enzyme activity analysis, 0.1 g of the frozen tissue samples were ground in the 0.9% NaCl solution with a weight/volume ratio of 1:9. The resulting mixture was subjected to centrifugation at 1500× *g* for 15 min at a temperature of 4 °C. The resulting supernatant was then transferred to a fresh centrifuge tube. Serum total antioxidant capacity (T-AOC), lactic dehydrogenase (LDH), acid phosphatase (ACP), and lysozyme (LZM) were measured. Furthermore, T-AOC, SOD, and CAT activities and the content of malondialdehyde (MDA) of the liver were measured. The enzyme activities were finally corrected through the protein concentration of samples. The protein concentrations were measured using the Enhanced BCA Protein Assay Kit (Beyotime Biotechnology, Beijing, China). The enzyme activities in this study were determined by the commercial assay kits provided by Nanjing JianCheng Bioengineering Institute in Nanjing, China, in accordance with the manufacturer’s instructions. 

### 2.4. Total RNA Extraction, cDNA Synthesis, and Real-Time Quantitative Polymerase Chain Reaction PCR (RT-qPCR)

The RNA iso Plus (Takara, Japan) was utilized to isolate the total RNA from the liver and head kidney. The concentration and quality of the extracted total RNA were determined using a NanoDrop^®^ 2000 spectrophotometer (Thermo Scientific, Waltham, MA, USA). Subsequently, the RNA that met the necessary criteria was reverse transcribed into complementary DNA (cDNA) utilizing the PrimeScript™ RT reagent Kit (Takara, Tokyo, Japan). The mRNA expression levels were quantified using the SYBR qPCR Master Mix (Takara) in the CFX96™ Real-Time System Thermal Cycler machine (BIO-RAD, Hercules, CA, USA). The RT-qPCR primer sequences for the target genes were designed using Primer Premier 5.0 software, based on the nucleotide sequences of the Japanese flounder (Table 1). These primers exhibited an amplification efficiency between 95% and 100%. The total volume of the RT-qPCR reaction was 20 μL, comprising 10 μL SYBR, 6 μL RNase-free water, 2 μL cDNA, and 1 μL each primer. The PCR protocol involved 39 cycles of a denaturation step at 95 °C for 10 s, an annealing step at 58 °C for 15 s, and an extension step at 72 °C for 10 s. *β-actin* was employed as the housekeeping gene based on our previous research [23]. The gene expression levels were calculated and normalized to *β-actin* using the 2^−ΔΔCT^ method [24].

### 2.5. Western Blot Analysis

In this study, the total protein extraction kit (Beyotime Biotechnology, Beijing, China) was used to extract the total proteins from the head kidney. To maintain uniformity, all the protein concentrations were equalized following the manufacturer’s instructions before denaturation. Subsequently, 20 μg protein samples were loaded into a 10% sodium dodecyl sulfate-polyacrylamide gel electrophoresis system. Polyvinylidene difluoride (PVDF) membranes (Merck Millipore, Germany) were utilized to transfer the proteins and then the membranes were incubated with non-fat milk for a duration of 2 h at room temperature. The membranes were then exposed to the targeting antibody overnight at 4 °C, followed by incubation with the secondary antibody (HRP-labeled Goat Anti-Rabbit IgG (H + L)) for 2 h at room temperature. Finally, we visualized the immune complex using the ECL Plus kit (Beyotime Biotechnology, Beijing, China) and analyzed the results using the ImageJ software 6.0 (National Institutes of Health, Bethesda, MD, USA). Primary antibodies used in this study were against following proteins according to previous studies: XBP1 (12782, CST, Boston, MA, USA), GRP78 (3177, CST), SAPK/JNK (9252, CST), phospho-SAPK/JNK (Thr183/Tyr185; 4668, CST), p38 (8690, CST), phospho-p38 (Thr180/Tyr182; 9215, CST), and β-actin (4970, CST) [23,25].

### 2.6. Calculations and Statistical Analysis

Survival rate (SR, %) = N_f_ × 100/N_i_ (1)

N_i_ and N_f_ were the initial and final numbers of fish, respectively.

Data analysis was conducted using SPSS 22.0 (IBM, Armonk, NY, USA). The Results are presented as the means with standard error of mean (S.E.M.), and the data were evaluated by independent samples *t*-test. All statistics performed with *p* < 0.05 were considered to be significant (* *p* < 0.05 and ** *p* < 0.01).

## 3. Results

### 3.1. Survival Rate

**Figure 1:**
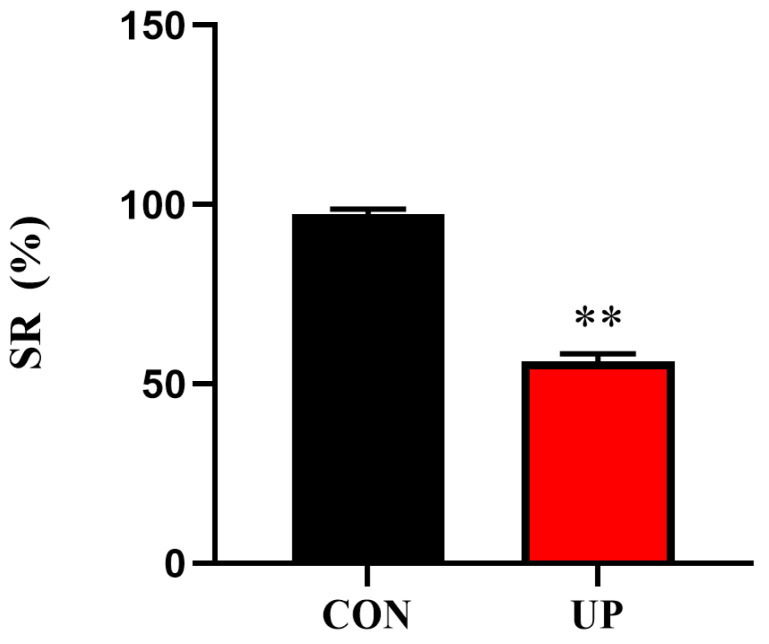
Effects of *Ulva prolifera* stress on the survival rate (SR) of the Japanese flounder. Results are presented as the means ± S.E.M. and were analyzed using independent samples *t*-test (*n* = 3). ** *p* < 0.01 indicates significant differences compared with the control group.

The results showed that *U. prolifera* stress significantly decreased the SR of the Japanese flounder compared with the control group (*p* < 0.01) (Figure 1).

### 3.2. Serum T-AOC Capacity and Non-Specific Immune-Related Enzyme Activities

**Figure 2:**
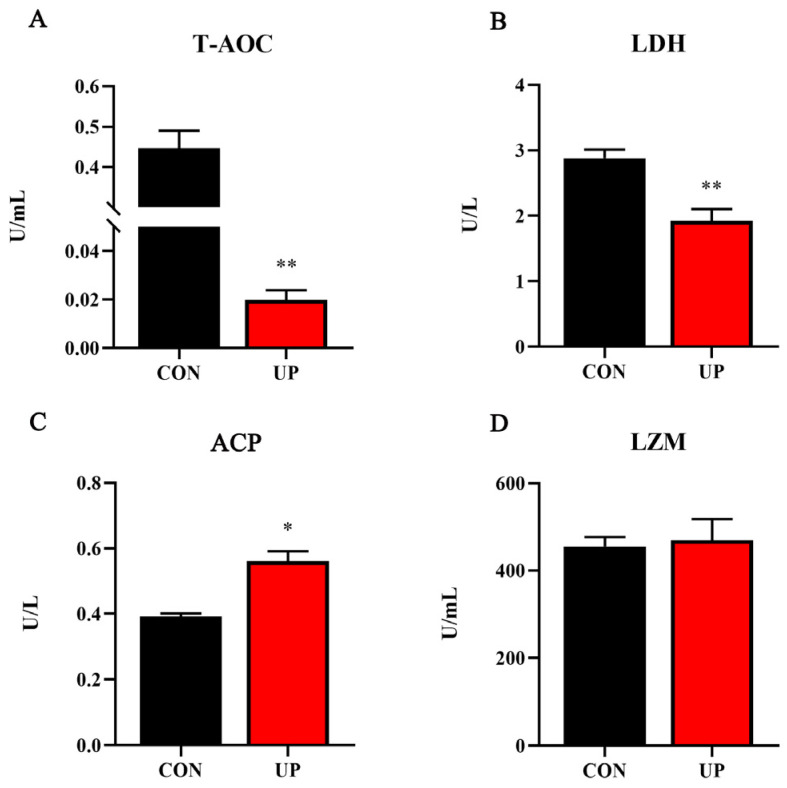
Effects of *U. prolifera* stress on serum T-AOC capacity and non-specific immune-related enzyme activities of the Japanese flounder. T-AOC—total antioxidant capacity (**A**); LDH—lactic dehydrogenase (**B**); ACP—acid phosphatase (**C**); LZM—lysozyme (**D**). Results are presented as the means ± S.E.M. and were analyzed using independent samples *t*-test (*n* = 4). * *p* < 0.05 and ** *p* < 0.01 indicate significant differences compared with the control group.

Then, serum T-AOC capacity and non-specific immune-related enzyme activities were detected. The results showed that T-AOC and LDH activities under *U. prolifera* stress conditions were significantly decreased compared with the control group (*p* < 0.05) (Figure 2A,B). Moreover, *U. prolifera* stress significantly increased the acid phosphatase (ACP) activity compared with the control group (*p* < 0.05) (Figure 2C). However, no significant difference in the activity of serum LZM was found among the two groups (*p* > 0.05) (Figure 2D).

### 3.3. Liver Antioxidant Capacity and Related Genes Expression

**Figure 3:**
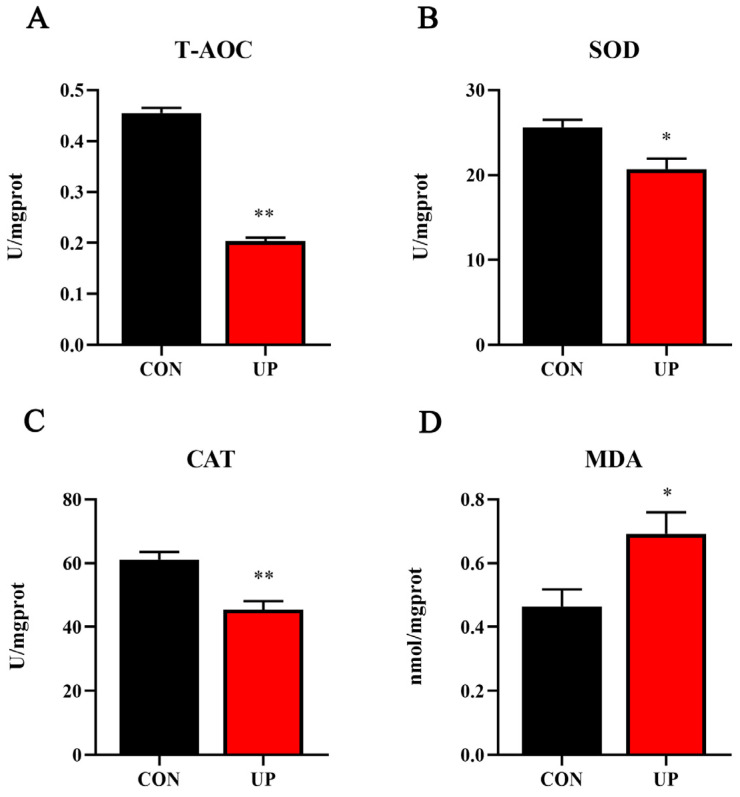
Effects of *U. prolifera* stress on the antioxidant capacity in the liver of the Japanese flounder. T-AOC—total antioxidant capacity (**A**); SOD—superoxide dismutase (**B**); CAT—catalase (**C**); MDA—the content of malondialdehyde (**D**). Results are presented as the means ± S.E.M. and were analyzed using independent samples *t*-test (*n* = 4). * *p* < 0.05 and ** *p* < 0.01 indicate significant differences compared with the control group.

**Figure 4:**
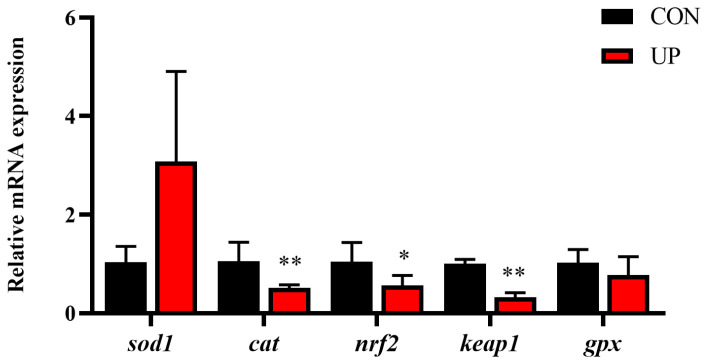
Effects of *U. prolifera* stress on the mRNA expression of antioxidant-related genes (*sod1*, *cat*, *nrf2*, *keap1* and *gpx*) in the liver of the Japanese flounder. Results are presented as the means ± S.E.M. and were analyzed using independent samples *t*-test (*n* = 4). * *p* < 0.05 and ** *p* < 0.01 indicate significant differences compared with the control group.

Effects of *U*. *prolifera* stress on antioxidant capacity in the liver of Japanese flounder were further detected. Results showed that T-AOC, SOD, and CAT activities of the liver under *U. prolifera* stress conditions were significantly decreased compared with the control group (*p* < 0.05), while the concentration of MDA was significantly increased in the liver compared with the control group (*p* < 0.05) (Figure 3). Then, the mRNA expressions of antioxidant-related gene (*sod1*, *cat*, *nrf2*, *keap1,* and *gpx*) were further examined. Results showed that *U. prolifera* stress significantly upregulated the mRNA expression levels of *cat*, *nrf2,* and *keap1* (*p* < 0.05), while there were no significant differences in the expression levels of *sod1* and *gpx* (*p* > 0.05) (Figure 4). The results of gene expressions were consistent with the trend of enzyme activities.

### 3.4. Head Kidney Inflammatory Genes and MAPK Signaling Pathway Expression

**Figure 5:**
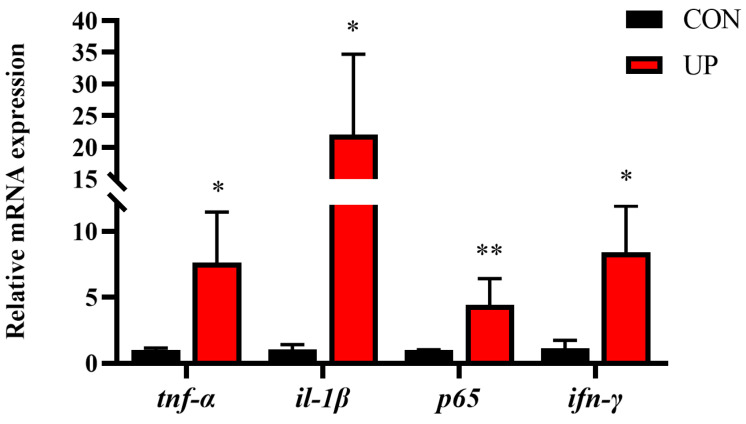
Effects of *U. prolifera* stress on the mRNA expression of inflammatory response-related genes (*tnf-α*, *il-1β*, *p65*, and *ifn-γ*) in the head kidney of the Japanese flounder. Results are presented as the means ± S.E.M. and were analyzed using independent samples *t*-test (*n* = 4). * *p* < 0.05 and ** *p* < 0.01 indicate significant differences compared with the control group.

**Figure 6:**
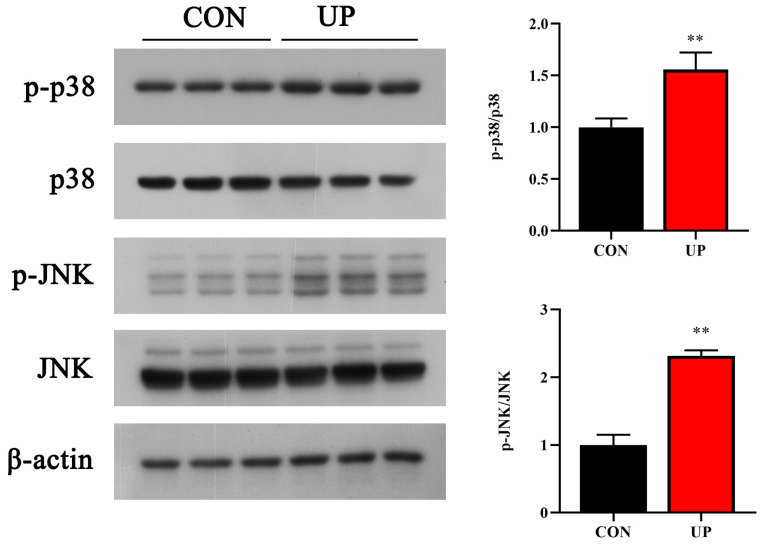
Effects of *U. prolifera* stress on the activation of p38 and JNK MAPK signaling pathways in the head kidney of the Japanese flounder. Results are presented as the means ± S.E.M. and were analyzed using independent samples *t*-test (*n* = 3). ** *p* < 0.01 indicates significant differences compared with the control group.

To understand the adverse effect of *U. prolifera* stress on the Japanese flounder, the inflammation related gene and MAPK signaling pathway expressions were detected. Results showed that *U. prolifera* stress significantly upregulated the pro-inflammatory gene expressions compared with the control group, including *tnf-α*, *il-1β*, *ifn-γ*, and *p65* (*p* < 0.05) (Figure 5). Moreover, the phosphorylation levels of MAPK pathway, including the JNK and p38, were significantly increased under *U. prolifera* stress conditions compared with the control group (*p* < 0.05) (Figure 6).

### 3.5. Head Kidney Endoplasmic Reticulum Stress-Related Genes and Proteins Expression

**Figure 7:**
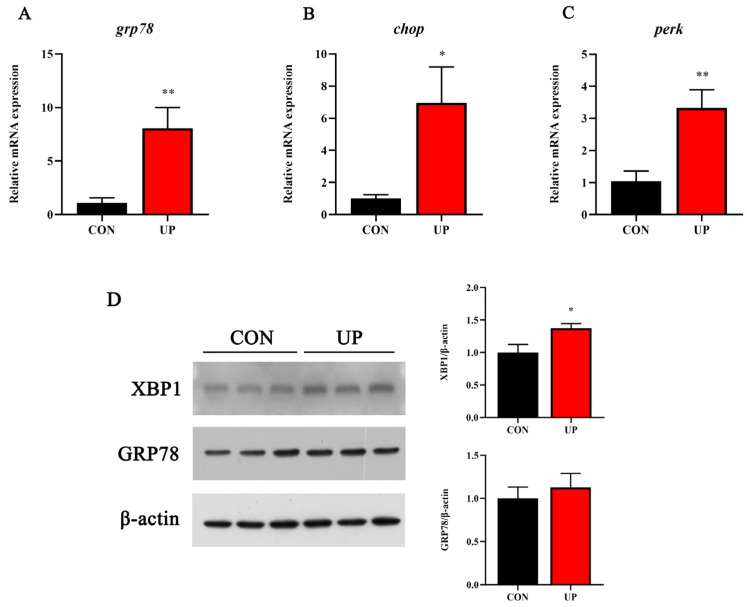
Effects of *U. prolifera* stress on the endoplasmic reticulum stress-related gene (*grp78*, *chop*, and *perk*) (**A**–**C**) and protein (XBP1 and GRP78) (**D**) expressions in the head kidney of the Japanese flounder. Results are presented as the means ± S.E.M. and were analyzed using independent samples *t*-test (*n* = 3). * *p* < 0.05 and ** *p* < 0.01 indicate significant differences compared with the control group.

The ER stress is closely related to inflammatory response. Thus, the ER stress-related gene and protein expressions were further detected. Compared to the control group, *U. prolifera* stress significantly increased the mRNA expression of URP-related genes, including *grp78*, *chop*, and *perk* in the head kidney (*p* < 0.05) (Figure 7A–C). Simultaneously, the protein expressions of XBP1 and GRP78 were significantly elevated under *U. prolifera* stress conditions (*p* < 0.05) (Figure 7D).

## 4. Discussion

*U. prolifera*, commonly known as green-tide-forming macroalgae, has increasingly become a focal point in the realms of marine environment protection and marine bio-resources, which is steadily gaining recognition in these fields [26]. In the present study, we firstly selected the intermediate size of the Japanese flounder (283.11 ± 6.45 g) and then investigated the effects of *U. prolifera* stress on the survival rate. The SR of Japanese flounder were significantly decreased, which was consist with the previous findings that massive macroalgal blooms caused the death of sea cucumber and shellfish in the Yellow Sea [10].

Based on the results of the growth performance, the effects of *U. prolifera* stress and degradation on the antioxidant capacity of the Japanese flounder were further explored. Previous studies have shown that the disruption of the antioxidant defense system can result in the generation of a vast array of oxidative intermediates, consisting of free radicals and oxygen-containing molecules that are non-free radicals [27]. These key antioxidant enzymes (T-AOC, MDA, SOD, and CAT) play crucial roles in regulating the antioxidant defense system. The SOD catalyzes the conversion of superoxide into H_2_O_2_, while CAT or GPx reduces H_2_O_2_ to H_2_O [28,29]. In the present study, the T-AOC activity of the serum and liver were significantly decreased during *U. prolifera* stress conditions accompanied by a significant increase in the MDA level in the liver. SOD serves as the first line, specifically catalyzing the dissociation reaction of superoxide anion to hydrogen peroxide and oxygen; subsequently, CAT eliminates hydrogen peroxide, thereby reducing its toxic effect [30]. Therefore, the observed decrease in SOD and CAT activities, and downregulated expressions of oxidative-stress-related gene during the 15-day exposure experiment suggested that *U. prolifera* stress could induce the liver oxidative stress, alter antioxidant capabilities, affect its physiological function and impair tissue structure of the Japanese flounder. A possible explanation is that the antioxidant system was exposed to *U. prolifera* conditions for a long time, releasing a lot of harmful substances causing the severe oxidative damage. The results were consistent with the previous finding in zebrafish embryos exposed to high concentration of *Microcysti* [31,32]. Similarly, previous studies in fish illustrated that renal oxidative damage is triggered by different stressors, leading to the generation of reactive oxygen species (ROS) [33,34]. The antioxidant defense mechanism is crucial for removing intracellular ROS [7]. *U. prolifera* stress may suppress the antioxidant capacity of fish due to the large consumption of oxygen and the enhanced cellular oxidative stress, resulting in low-oxygen or even hypoxic conditions in the coastal water [35].

The innate immune system of fish is widely considered as the first line of defense against various environmental stress, such as pathogens, hypoxia, and heavy metal ions [19,36,37]. In addition to oxidative stress, *U. prolifera* stress could induce fish inflammatory response and alter immunity levels by the non-specific immunity and other immune elements. Consequently, it is greatly crucial to clarify the effects of *U. prolifera* stress on inflammatory gene and related pathway expressions. In this study, the pro-inflammatory gene (*tnf-α*, *il-1β*, *ifn-γ*, and *p65*) levels were all strongly upregulated and the MAPK pathway was induced under *U. prolifera* conditions, which suggested that *U. prolifera* degradation had an activation effect on the inflammatory response. However, the prolonged inflammatory response in *U. prolifera* conditions could eventually lead to the irreversible tissue damage, reduced immunity among the fish, and potentially death [38]. Previous studies have proved that *U. prolifera* stress significantly affects the genes and signaling pathways related to immunity and metabolism in the intestines [22]. These results were also consistent with the effects of hypoxia on the immune regulation observed in Nile tilapia and Atlantic salmon [39]. Moreover, the research has suggested that the UPR is interconnected with inflammatory response through various mechanisms, including the production of ROS, inflammatory genes, and the MAPK pathway [40,41]. In this study, *U. prolifera* degradation significantly elevated the expression of endoplasmic reticulum stress-related genes and proteins, which was firstly proved in marine fish. Among the three major UPR transducers, the PERK and XBP1 pathways also were influenced under *U. prolifera* stress conditions.

As the global temperature rises and seawater eutrophication intensifies, the prevalence of macroalgal blooms, particularly *Ulva prolifera*, is becoming an increasingly pressing concern. While *U. prolifera* itself is non-toxic, its negative effects primarily stem from the harmful substances and water pollution resulting from its overgrowth and degradation. Decaying *U. prolifera* releases large amounts of hazardous substances; this not only affects aquatic life in the sea but also has a huge impact on aquaculture [42,43,44]. The natural decomposition of *U. prolifera* releases substantial quantities of the biogenic elements, such as carbon (C), nitrogen (N), phosphorus (P), sulfur (S), and metallic elements like iron (Fe) [45]. These released elements could alter the chemical properties of the local seawater, resulting in hypoxia in the environment. Subsequently, such changes further foster microbial proliferation, thereby disrupting the survival of marine organisms. Therefore, coastal hypoxia and acidification may occur due to the rapid microbial respiration triggered by macroalgae decomposition [6]. Meanwhile, the decomposition of macroalgae may also lead to eutrophication by releasing carbon, nitrogen, and phosphorus into the environment [11,46]. Although the effects of *U. prolifera* on the antioxidant capacity and inflammatory response have been explored in the present study, the molecular mechanism required further investigation. On the other hand, previous studies have found that polysaccharides extracted from marine algae, such as *U. prolifera*, had positive effects in regulating various biological activities [47,48]. Thus, optimizing the utilization of *U. prolifera* extraction could be a vital strategy in solving the harmful effects of *U. prolifera* overgrowth, which also requires further study.

## 5. Conclusions

In conclusion, this study revealed that *U. prolifera* stress could reduce the survival rate, suppress antioxidant capacity in the liver, and induce an inflammatory response in the head kidney of Japanese flounder. This study could advance our understanding of the adverse effects of *U*. *prolifera* stress on marine benthic fish and promote the sustainable development of aquaculture.

## Figures and Tables

**Table 1:** Primers used for RT-qPCR and genes accession number.

Gene	Forward (5′–3′)	Reverse (5′–3′)	Accession Number
*β-actin*	AGGTTCCGTTGTCCCG	TGGTTCCTCCAGATAGCAC	XM_020103099
*tnf-α*	GTCCTGGCGTTTTCTTGGTA	CTTGGCTCTGCTGCTGATTT	XM_020104959
*il-1β*	CTGTCGTTCTGGGCATCAAA	AACAGAAATCGCACCATCTCACT	XM_020105656
*ifn-γ*	AGTGGTCTGTCTGTCCCTGTG	GCTTCCCGTTGAATCTGTCTT	AB435093
*p65*	GCTTCTCTGGGTAGCACACC	GGGTTCAGAAGGTCCACAAA	XM_020100108
*sod1*	CGTTGGAGACCTGGGGAATGTG	ATCGTCAGCCTTCTCGTGGATC	EF681883
*cat*	CACGGACCAGATGAAGCAGTG	CCTTGGAGTAGCGGGTAATGTC	XM_020079314
*nrf2*	GAAGAACAAGGTGGCGGCTCAG	GAAGGTCAGGCTGTGCTGGAAC	XM_020096126
*keap1*	GGAGCCGTGCCAGAAAGAAGTG	GTGCCGCTGACTGTGGTGAAC	XM_020084284
*gpx*	GGTGGATGTGAATGGGAAGGATGC	TTGTATCGTCGCTGGGAAATGGC	EU095498
*grp78*	GTCGTGAGGTTGAGAAGGCA	TCATGGTGGAACGGAACAGG	DQ662232
*chop*	CGGCCAAAAAGAGTCGCAAA	TCTCCGCTTTCAATCGCTCA	XM_020096956
*perk*	CTACCACCTACATCGTCCGC	ACCGGCTCAAAGTCAGTCAG	XM_020105998

## Data Availability

Data is contained within the article.

## Supplementary Materials

The following supporting information can be downloaded at: https://www.mdpi.com/article/10.3390/ani13243768/s1, Figure S1: Graphics for the experimental design.
