# Supplementary figures and images for: Ulva prolifera Stress in the Yellow Sea of China: Suppressed Antioxidant Capacity and Induced Inflammatory Response of the Japanese Flounder (Paralichthys olivaceus)
Source: Animals (Basel). 2023 Dec 6;13(24):3768. doi: 10.3390/ani13243768 (PMC10741151; doi:10.3390/ani13243768)

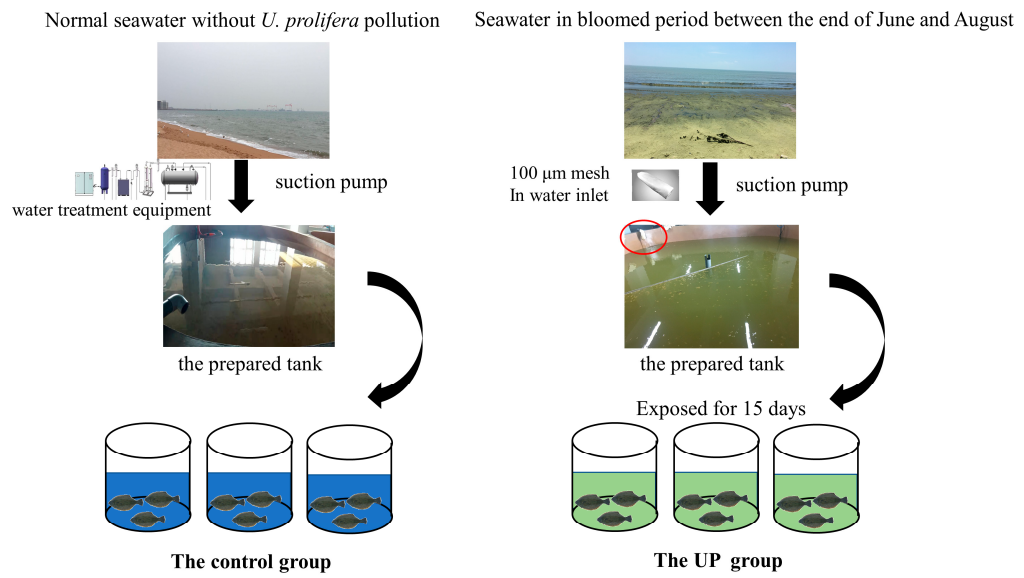

**Figure S1.** Graphics about the experimental design

Supplement: Supplementary file 1 [file animals-13-03768-s001.zip › animals-2722277-supplementary.pdf]
